# Improvement of the Lyophilization Survival Rate of *Lactobacillus casei* via Regulation of Its Surface Substances

**DOI:** 10.3390/foods11213468

**Published:** 2022-11-01

**Authors:** Shumao Cui, Ziyi Pan, Sheng Wu, Bingyong Mao, Xin Tang, Qiuxiang Zhang, Hao Zhang, Jianxin Zhao

**Affiliations:** 1 State Key Laboratory of Food Science and Technology, Jiangnan University, Wuxi 214122, China; 2 School of Food Science and Technology, Jiangnan University, Wuxi 214122, China; 3 National Engineering Research Center for Functional Food, Jiangnan University, Wuxi 214122, China

**Keywords:** lyophilization survival rate, surface substance, *Lactobacillus casei*, fermentation conditions

## Abstract

The influence of surface substance production on the freeze-drying survival of *Lactobacillus casei* and methods to control the surface substances during fermentation were studied. The bacteria were treated with hypertonicity combined with ultrasound, and the survival rate was determined. The optimal conditions for removing surface substance without harming the bacteria were 81 w/18 min. The surface substances provided a protective effect on the lyophilization of the bacteria without protectants. However, in the presence of protectants, excessive surface substances reduced the protective effect of the optimum protectant alginate to 39.69 ± 1.27%. Finally, the amount of surface substances and lyophilized survival rate of collected bacteria were determined by adding EDTA during fermentation and regulating fermentation conditions, such as the carbon source, carbon-to-nitrogen ratio, and pH. The highest survival rate was 85.79 ± 3.29%, which was achieved when the amount of surface substances was (2.82 ± 0.55) × 10^−11^ mg/CFU. Therefore, the production of surface substances by the bacteria could be reduced by modifying the fermentation stage, which has significance in the improvement of the lyophilization survival rate of *L. casei* and the number of live bacteria per unit mass of *L. casei* in the lyophilized preparation.

## 1. Introduction

During growth and metabolic activity, *Lactobacillus casei* generates surface substances such as exopolysaccharides and proteins [1,2]. Exopolysaccharides mainly exist in two forms: mucopolysaccharides secreted into the culture medium and capsular polysaccharides (CPSs) attached to the surface of the bacteria [3]. Capsular polysaccharides are attached to the cell through the hydroxyl structure. Because of this particular structure, the hydroxyl group tends to form hydrogen bonds with water molecules, leading to a higher water content per gram of wet bacteria mud [4] and a lower viable count [5]. Surface proteins (SPs) include the S-layer, membrane, and other secreted proteins [6]. As metabolic products of bacteria, CPSs and SPs communicate information with the external environment. Capsular polysaccharides can be used as pathogenic factors in many microorganisms to enhance the infectivity of pathogenic bacteria. Bacterial SPs are also essential for information exchange between microorganisms and the external environment [7].

Numerous studies have shown that surface substances produced by lactobacilli are functional substances that afford protection from harsh environments [8]. Exopolysaccharides could improve resistance to dry environments and phagocytosis by other cells [9], resistance to antibiotics, osmotic pressure, resistance to phage attack [10], and the freeze-dried survival rate [11]. Dimopoulou et al. found that the survival rate of *Oenococcus oeni*-producing CPSs is significantly higher than that of *Oenococcus oeni*-not producing CPSs, which indicated that the CPSs produced by *Oenococcus oeni* could protect the cell structure in cooperation with maltodextrin or sucrose, thereby improving the freeze-drying survival rate [12]. Effendi et al. also showed that exopolysaccharides of the Antarctic cyanobacterium *Nostoc* sp. strain SO-36 could adapt to harsh environments, such as low temperature [13]. SPs play an important role in bacterial adhesion and have a molecular sieve function, which affects the freeze-drying effect of bacteria via the material exchange between the bacteria and the environment. However, excessive surface substance production makes it more difficult to collect bacteria mud [5] and could impact the effect of lyophilized protectants. At present, it remains unknown whether there is a difference in the lyophilization protection effect between CPSs and lyophilized protective agents.

The surface matter yield of lactobacilli differed for various sources. Exopolysaccharide production was influenced by factors other than differences in the strain and extraction methods. Culture conditions are the main factors affecting extracellular polysaccharide production [14], including fermentation substrates, such as carbon sources, nitrogen sources, micronutrients, salt ions, culture temperature [15], and pH [16]. The carbon source has the greatest influence [17]. Temperature and other conditions significantly impact the production and yield of surface substances by bacteria [18]. Marion et al. studied the effects of different sugars on the viscosity production characteristics of *L. brevis* TMW 1.2112. They found that when hexose was used as a fermentation substrate, it could significantly promote the formation of extracellular polysaccharides in bacteria and improve the viscosity of the fermentation liquid and bacteria [19]. However, Oliveira et al. found that EPS production was higher with soybean molasses-based-medium than with hexose medium [20].

At present, most studies have shown that surface substances are conducive to improving the freeze-drying survival rate of lactobacilli. However, we previously found that excessive surface substances will reduce the freeze-drying survival rate. It will be important to reveal the relationships between surface substance content and freeze-drying survival rate. This study aims to investigate the specific impact of surface substances on the freeze-drying process of lactobacilli and explore its mechanism. The freeze-drying survival rate of lactobacilli can be improved by changing fermentation conditions to regulate surface substances at appropriate content. This is helpful in guiding lactobacilli production by improving the freeze-drying survival rate and the viable number of lactobacilli per unit mass.

## 2. Materials and Methods

### 2.1. Microorganisms and Culture Conditions

*Lactobacillus casei* FJSWX3-L3 was isolated from healthy human feces and stored at the Culture Collection of Food Microorganisms (CCFM) at Jiangnan University. This strain was cultured in MRS broth and incubated at 37 °C under anaerobic conditions.

### 2.2. Surface Substance Stripping Method

*L. casei* FJSWX3-L3 was cultivated for 12 h and centrifuged at 8000× *g* for 15 min. Bacterial cells were collected, washed twice in saline, and resuspended in 1 M NaCl solution. Then, surface substances, including CPSs and SPs, were stripped with ultrasonic treatment [21]. By adjusting the operating frequencies and times, the most appropriate conditions were determined in which the bacteria could survive well, and their surface substance would be stripped completely. The working frequency of ultrasonic treatment was 63 W/72 W/81 W/90 W and the working times were 6 min, 12 min, and 18 min. The number of viable bacteria was determined using the plate counting method. The survival rate was calculated as the viable number before and after the surface substance was stripped.

### 2.3. Capsule Observation and Determination

So as to visualize and identify whether the bacterial cell capsule was completely stripped, 10 μL of the cell suspension was deposited on a lame, prefixed with 4% glutaraldehyde (Shanghai Aladdin Bio-Chem Technology Co., Ltd., Shanghai, China), and fixed with 2% osmium tetroxide (Sinopharm Chemical Reagent Co., Ltd., Shanghai, China) at room temperature. The fixed samples were then gradually dehydrated in 75% ethanol (Sinopharm Chemical Reagent Co., Ltd., Shanghai, China), embedded in epoxy resin, and prepared as ultrathin sections. Double staining was performed using uranium-dioxyacetate and lead citrate (Shanghai Acmec Biochemical Co., Ltd., Shanghai, China). The samples were then observed using transmission electron microscopy (TEM)(Hitachi, Ltd., Tokyo, Japan).

### 2.4. Freeze-Dried Bacterial Cell Preparation with Physiological Saline Solution

To detect the protection of the bacterial surface substance during the freeze-drying process, after the surface substance was stripped, the cell suspension was immediately centrifugated (8000× *g*, 15 min). The supernatant was collected to determine the surface substance content, which contained CPSs and SPs. The stripped cells were resuspended in physiological saline solution at a 1:1 (*m*/*v*) ratio and vortexed gently. One milliliter of the bacterial suspension was placed in a glass bottle for subsequent lyophilization.

### 2.5. Lyophilized Bacterial Cell Pellets with Different Cryoprotectants

In order to analyze the effects of various anti-freeze agents in the autologous bacterial capsule and exogenous carbohydrate protectant, various cryoprotectant agents were applied to protect bacterial survival. Stachyose, trehalose, and synanthrin (Shanghai Canspec Scientific Instruments Co., Ltd., Shanghai, China) were selected to prepare a 20% (*m*/*v*) protectant solution to mix with the bacteria at a ratio of 1:1 (*m*/*v*), wherein 1 g bacterial mud was mixed with 1 mL cryoprotectant agent. The control group was an *L. casei* suspension with no treatment, and the test groups were named the *L. casei* stripped surface substance. The cell suspension was homogenously mixed with the four different cryoprotectants, and the cell suspension was maintained at −20 °C for subsequent lyophilization. 

### 2.6. Determination of the Content of the Surface Substances of L. casei

Single-cell surface substances mainly included CPSs and SPs, whereas lipopolysaccharides and lipooligosaccharides were common in gram-negative bacteria. In addition, SPs were common for lactobacilli. The amount of CPSs was determined using phenol sulfuric acid (Sinopharm Chemical Reagent Co., Ltd., Shanghai, China), whereas SP was determined with the Kjeldahl method following the Chinese detection standard. The viable number per gram of bacterial mud was then determined to calculate the surface substance content for each cell. The surface substance content was calculated using the following formula:

$$\mathrm{Total}\;\mathrm{content}\left(\mathrm{mg}/\mathrm{CFU}\right)=\frac{\mathrm{CPS}\;\mathrm{content}}{\mathrm{bacteria}\;\mathrm{mud}\;\mathrm{mass}\times \mathrm{viable}\;\mathrm{number}\;\mathrm{per}\;\mathrm{gram}}+\frac{\mathrm{protein}\;\mathrm{content}}{\mathrm{bacteria}\;\mathrm{mud}\;\mathrm{mass}\times \mathrm{viable}\;\mathrm{number}\;\mathrm{per}\;\mathrm{gram}}$$

### 2.7. Cultivation with Different Carbon Sources and Lyophilization

According to Fraunhofer et al., most pentoses failed to induce slime formation, indicating that fewer CPSs were produced using pentose during fermentation [19]. In contrast, the growth of all hexoses (d-glucose, d-fructose, and d-galactose) and disaccharides (d-maltose and d-melibiose) resulted in slimy cultures. Based on the above studies, different pentoses and hexoses were applied to explore the influence of carbon sources on the number of CPSs. Three cultivation media were used that utilized 20 g/L arabinose, lactose, or glucose (Shanghai Canspec Scientific Instruments Co., Ltd., Shanghai, China) as three different carbon sources and 25 g/L yeast extract FM 528 (Angel Yeast Co., Ltd., Yichang, China) as the nitrogen source. The other components were the same as those used for MRS broth. *L. casei* was inoculated into the three media at 2% (*v*/*v*). After cultivation, *L. casei* was prepared for capsule observation using TEM and lyophilized following the method described above. 

### 2.8. Cultivation with EDTA and Lyophilization

Ethylenediamine tetraacetic acid (EDTA) (Shanghai Canspec Scientific Instruments Co., Ltd., Shanghai, China)is commonly considered a divalent cation chelating agent, and the divalent cation is vital during the synthesis of CPSs [19]. Emma researched the inhibition of the growth of capsules of *Cryptococcus neoformans* and found that EDTA could effectively reduce the amount of the CPS glucuronoxylomannan shed into the biofilm matrix [22]. To evaluate if EDTA could effectively reduce the production of *L. casei* surface substance, 3.0 g/L and 1.5 g/L EDTA were added to the MRS broth, and MRS broth without any additions was used as the control group. *L. casei* was centrifuged to collect the cells and washed twice with saline solution. The cell pellets were resuspended in 20% (*w*/*v*) stachyose solution at a ratio of 1:1 (*m*/*v*) for lyophilization. The remaining cells were used for TEM observations.

### 2.9. Determination and Lyophilization of Surface Substances of Bacteria under Different Fermentation Conditions

#### 2.9.1. Surface Substance Content and Lyophilization Survival Rate of *L. casei* at a Constant pH with Different Carbon Sources

Three types of fermentation media with various carbon sources were prepared using glucose, arabinose, and lactose. The concentration of the carbon sources was 20 g/L, the attention of the nitrogen source (yeast extract FM 528) was 15 g/L, and inorganic salts and trace elements were the same as those in the MRS medium. The strains were activated according to the conditions in Section 2.1, inoculated with a 2% (*v*/*v*) inoculation in 5 L bioreactors (BIOTECH-5JS-3, Shanghai BaoXing Bio-Engineering Equipment Co., Ltd., Shanghai, China) (liquid volume was 3 L) at pH 6.0 and 37 °C, and collected during the logarithmic and stable phase, respectively. A part of the bacterial sludge was used to determine the surface substance content per unit of bacteria. A portion of the bacterial sludge was lyophilized by adding 200 g/L stachyose at a ratio of 1:1 (g:mL), and the number of viable bacteria before and after lyophilization and the survival rate were calculated.

#### 2.9.2. Surface Substance Content and Lyophilization Survival Rate of *L. casei* at a Constant pH with Different Nitrogen Sources

Different nitrogen sources (e.g., soy peptone, yeast extract FM528, and tryptone (Angel Yeast Co., Ltd., Yichang, China) were added to the medium to yield exact concentrations of 25.0 g/L, referring to the MRS medium for other nutrients. The strains were activated according to the conditions in Section 2.1 and inoculated with 2% (*v*/*v*) in 5 L bioreactors. See Section 2.9.1 for strain culture, collection methods, and lyophilization conditions.

#### 2.9.3. Surface Substance Content and Lyophilization Survival rate of *L. casei* at a Constant pH with Different Carbon–Nitrogen Consumption Ratios

A 3 L fermentation medium with carbon-to-nitrogen (C/N) ratios of 4:1 and 4:3 was prepared. The nitrogen source was yeast extract FM 528 with a glucose mass concentration of 80 g/L, and the other nutrients were those added to the MRS medium. The strains were activated according to the conditions in Section 2.1, inoculated with 2% (*v*/*v*) in 5 L bioreactors. See Section 2.9.1 for strain culture, collection methods, and lyophilization conditions.

#### 2.9.4. Surface Substance Content and Lyophilization Survival Rate of *L. casei* at Different pH

The C/N ratio was 4:3, the carbon source was 80 g/L glucose, and the nitrogen source was FM528. Subsequently, batch culture was performed at a controlled pH of 5.0 or 6.5. See Section 2.9.1 for the strain culture, collection methods, and lyophilization conditions.

### 2.10. Viable Counting Method

Colony counting was performed to calculate the cell survival rate after freeze-drying. Lyophilized powders collected under different fermentation conditions were suspended in a sterile saline solution and gently vortexed for 10 s. Serial dilutions were prepared from the initial suspensions and cultivated on MRS agar. Each sample was tested thrice. Agar plates were incubated at 37 °C for 72 h under anaerobic conditions. The results were expressed as log colony-forming units per gram (log CFU/mL).

### 2.11. Statistical Analysis

The results of three independent experiments were tested, and statistical analysis was performed using SPSS 20.0 (SPSS Inc., Chicago, IL, USA) and GraphPad Prism 6 (GraphPad Software LLC., San Diego, CA, USA). Significant differences were evaluated using a *t*-test analysis, and values were deemed significant when the *p* value was < 0.05.

## 3. Results

### 3.1. Effects of Surface Substances and Lyophilized Protectants on the Survival Rate of Lyophilization

**Figure 1:**
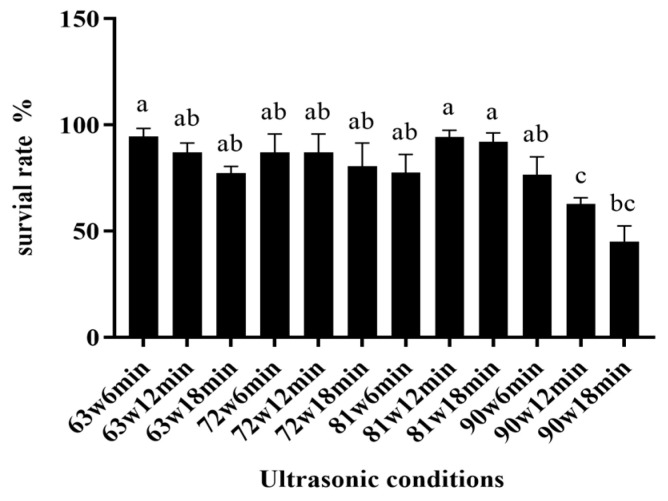
The survival rate of *L. casei* JSWX3-L3 in different ultrasonic conditions. Different letters indicate significant differences (*p* < 0.05).

In order to identify the function of surface substances, the conditions for stripping surface substances were optimized by setting different stripping conditions. So as to efficiently strip the surface substance of *L. casei* without damaging the activity of the bacteria, the cells were resuspended in 1 M NaCl solution. Because of the high osmotic pressure, the bacterial cells shrunk, the gap between the surface material and cell wall increased, and the surface substance was more likely to be broken and dissolved under the action of ultrasonic cavitation. The results are shown in Figure 1. At lower power (63 W or 72 W), the cell activity was not affected, and there was no loss of cell activity even when the ultrasonic time was extended; however, with an increase in ultrasonic power, the tolerance of different strains showed differences. After the ultrasonic power exceeded 81 W, the survival rate of *L. casei* decreased with the extension of ultrasonic time, which was consistent with the optimized ultrasonic conditions for stripping the surface substances of probiotics. Racioppo et al. (2017) studied the effect of hydrophobicity and other properties of probiotics [23]. The optimal conditions for the surface substance stripping of *L. casei* JSWX3-L3 were 81 w/18 min.

**Figure 2:**
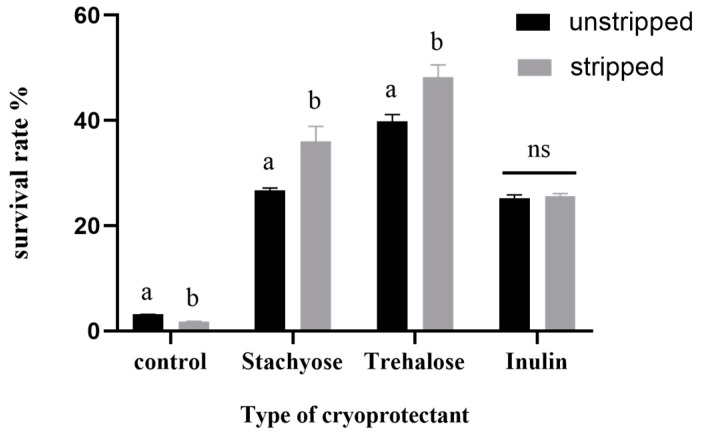
The survival rate of *L. casei* JSWX3-L3 before (left bars) and after (right bars) the surface substance stripped. Different letters indicate significant differences (*p* < 0.05).

It is widely thought that the surface substances of *L. casei* play an essential role during the *L. casei* lyophilization process, assisting the bacteria in surviving the adverse environment better. In our study, exopolysaccharides were beneficial to the survival of *L. casei* with rough freezing without cryoprotectants. The strains showed a lower survival rate after the surface substances were stripped (Figure 2), indicating that the surface substances indeed protected freeze-dried cells; however, the death rate was more than 95%, indicating that the protection of surface materials without the addition of cryoprotectants was not enough to reduce damage caused by lyophilization significantly.

Different cryoprotectant agents were added to the cell pellets to identify further differences in the protective effects of autologous surface substances and exogenous cryoprotectant agents. The freeze-drying survival rates are shown in Figure 2. When trehalose or stachyose was used as lyophilized protectants, the survival rate of *L. casei* stripped of surface substances was significantly higher than that of *L. casei* with surface substances. However, there was no significant difference in survival rate when inulin was used as a lyophilized protectant.

### 3.2. Effects of Adding EDTA on Surface Substance Yield and Survival Rate after Freeze-Drying

**Figure 3:**
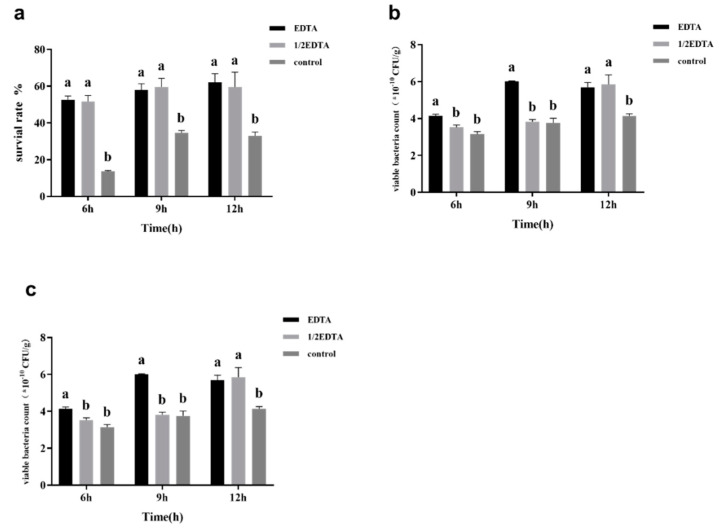
The lyophilized survival rate (**a**), surface substance content (**b**), and the number of viable counts (**c**) of *L. casei* FJSWX3-L3 during batch culture. Different letters indicate significant differences (*p* < 0.05).

In order to explore whether EDTA can reduce the production of surface substances and further reveal the relationship between the production of surface substances and the freeze-drying survival rate, EDTA was added to the MRS broth at a concentration of 3.0 g/L and 1.5 g/L. MRS broth without any additions was used as the control. As shown in Figure 3, EDTA addition during fermentation can effectively reduce the content of surface substances and lead to an approximately 2-fold increase in the freeze-drying survival rate. Although it is more effective for EDTA to reduce the content of surface substances at a concentration of 3.0 g/L than 1.5 g/L, there is no significant difference in the freeze-drying survival rate between 3.0 g/L and 1.5 g/L EDTA. Thus, EDTA should be added at a concentration of 1.5 g/L during the culture process. 

**Figure 4:**
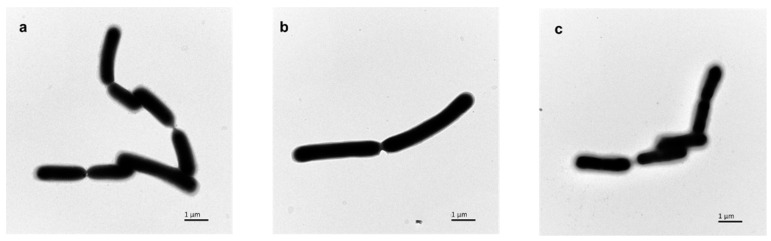
The morphology of *L. casei* FJSWX3-L3 under different culture conditions. (**a**) Cultured with arabinose; (**b**) cultured with EDTA addition; (**c**) cultured in MRS medium.

Images of the different morphologies of *L. casei* under different fermentation conditions were taken using TEM and are shown in Figure 4. In Figure 4c, there is an obvious floc on the surface of *L. casei* cultured in MRS medium, which is probably the surface substance of *L. casei* FJSWX3-L3. In Figure 4a,b, there is only a small amount of floc on the surface of the cell, indicating that using arabinose as the carbon source and the EDTA addition can effectively reduce the surface substances production of *L. casei* FJSWX3-L3. The freeze-drying survival rate was 62.06 ± 4.75% at batch culture with EDTA addition, which was significantly higher than that in the MRS medium.

### 3.3. Effects of Fermentation Conditions on Surface Substance Yield and Survival Rate during Freeze-Drying

**Figure 5:**
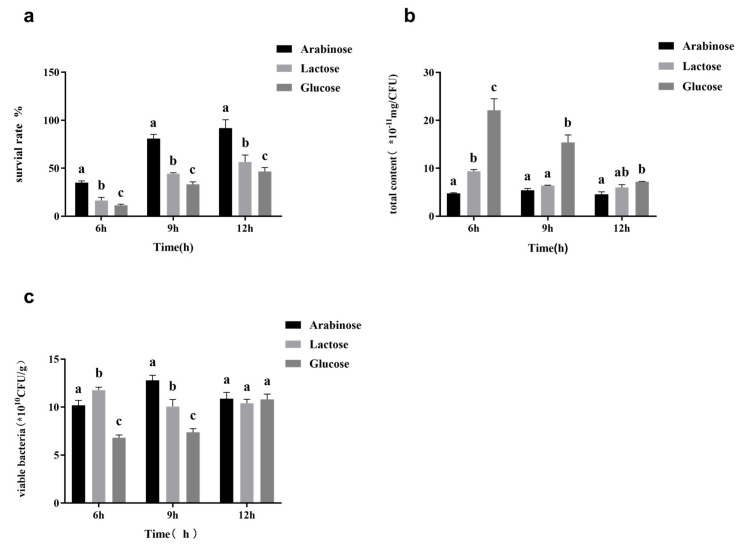
The lyophilized survival rate (**a**), surface substance content (**b**), and the number of viable counts (**c**) of *L. casei* FJSWX3-L3 cultured with different carbon sources. Different letters indicate significant differences (*p* < 0.05).

In order to further identify the relationship between the amount of surface substances and freeze-drying survival rate when using oligos as cryoprotectants, different carbon sources were added to the cultivation medium to determine the effect of the carbon source on the production of surface substances and freeze-drying survival rates. The type of carbon source significantly affected the production of the exopolysaccharides, and lactobacilli could not phosphorylate pentose to produce glucose 6-phosphate to participate in the exopolysaccharide pathway. A higher survival rate was observed with arabinose as the carbon source (Figure 5). The bacteria fermented with arabinose possessed a smaller amount of surface substances than those fermented with lactose and glucose, and bacteria fermented with arabinose showed a higher survival rate after freeze-drying. Thus, *L. casei* harvested during the same fermentation period showed a higher freeze-drying survival rate when it had a smaller amount of surface substances.

**Figure 6:**
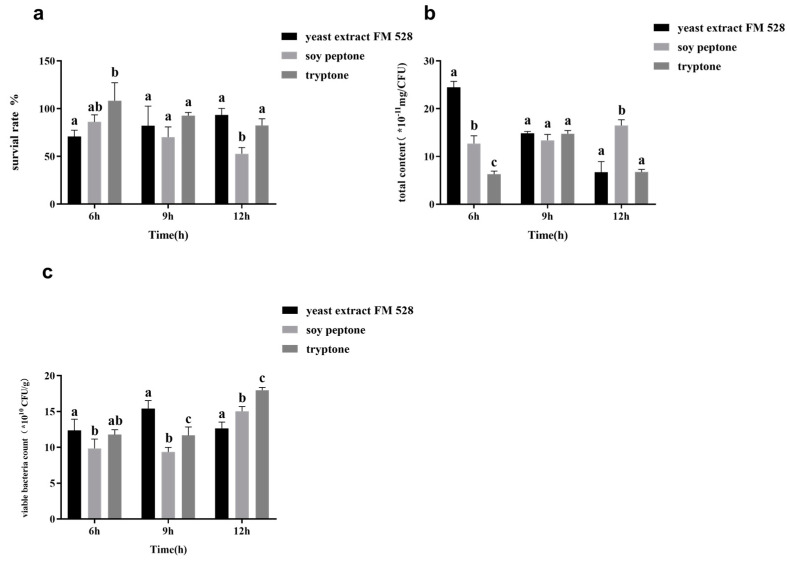
The lyophilized survival rate (**a**), surface substance content (**b**), and the number of viable counts (**c**) of *L. casei* FJSWX3-L3 cultured with different nitrogen sources. Different letters indicate significant differences (*p* < 0.05).

The proliferative effect of different nitrogen sources was preliminarily evaluated to study the effects of nitrogen sources on the surface substance yield and freeze-dried survival rate of lactobacilli. The nitrogen utilization characteristics of *L. casei* were studied by selecting plant and microbial nitrogen sources to ensure that the bacteria could proliferate, as shown in Figure 6. The lyophilized survival rate of *L. casei* FJSWX3-L3 cultured with tryptone and stachyose as protective agents was significantly higher than that using soybean peptone and yeast extract FM 528 during the logarithmic growth period (6 h). For *L. casei* FJSWX3-L3 at the stable stage, the lyophilized survival rate of the tryptone and yeast extract FM 528 groups was higher, and the surface substance content was significantly lower in the tryptone group than in the soybean peptone group. It appeared that the effect of surface substance production on the lyophilization survival rate was significant.

**Figure 7:**
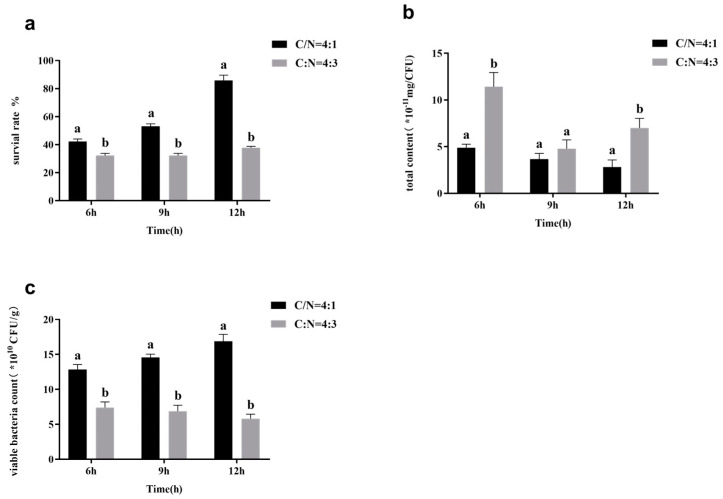
The lyophilized survival rate (**a**), surface substance content (**b**), and the number of viable counts (**c**) of *L. casei* FJSWX3-L3 cultured with different carbon–nitrogen ratios. Different letters indicate significant differences (*p* < 0.05).

Current studies have shown that a high C/N ratio in the culture medium could increase the production of microbial surface substances [24]. Culture media with different C/N ratios were used to culture *L. casei* FJSWX3-L3 at a constant pH to determine the production of surface substances and the freeze-dried survival rate of *L. casei* FJSWX3-L3 in the logarithmic and stationary phases. As shown in Figure 7, *L. casei* FJSWX3-L3 cultured at a low C/N ratio of 4:3 during the logarithmic growth stage (6 h) produced less surface substance content per unit of bacteria with a higher C/N ratio of 4:1, and a freeze-dried survival rate of 42.20 ± 1.57% in the low carbon–nitrogen group at 6 h, compared to 32.20 ± 1.35% in the high carbon–nitrogen culture. The lower production of surface substances was beneficial to the lyophilization survival of logarithmic *L. casei*. The number of viable bacteria per unit mass of wet slime obtained by low C/N ratio fermentation was significantly higher than that obtained in the high C/N ratio culture, which also confirmed that the lower the surface material, the higher the number of viable bacteria per unit mass of wet slime, and the highest survival rate of 85.79 ± 3.29% was achieved when the surface substance content was (2.82 ± 0.55) × 10^−11^ mg/CFU.

**Figure 8:**
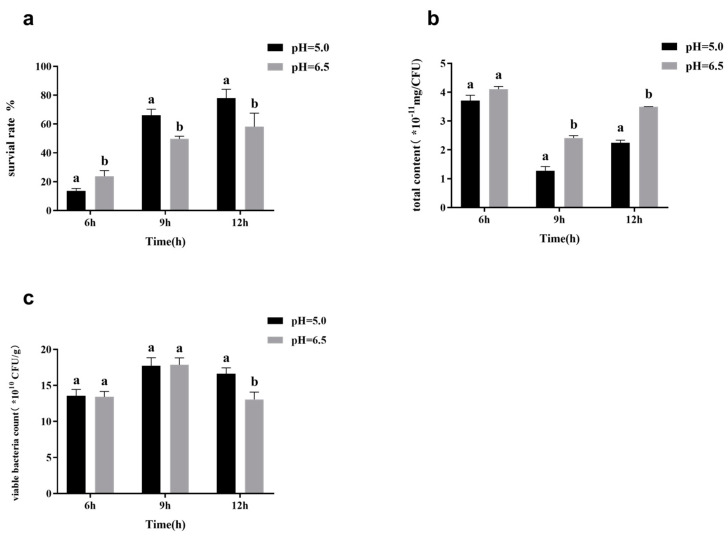
The lyophilized survival rate (**a**), surface substance content (**b**), and the number of viable counts (**c**) of *L. casei* FJSWX3-L3 cultured at different pH conditions. Different letters indicate significant differences (*p* < 0.05).

The growth and metabolism of lactobacilli are affected by various external environmental factors, among which pH is one of the most essential factors that mainly affects the activities of related enzymes and proteins in the metabolic process [25]. To study the influence of pH on the surface material yield and freeze-drying survival rate of *L. casei*, it was cultured at a constant pH and collected for freeze-drying, as shown in Figure 8. At the end of the logarithmic growth (9 h) and stable periods (12 h), there were significant differences in the surface substance content of *L. casei* FJSWX3-L3 under different pH values and in the freeze-dried survival rate of *L. casei* FJSWX3-L3 under the two cultures. *L. casei* had a higher surface substance content at the logarithmic inflection point (9 h) and stable stage (12 h) but a significantly lower freeze-dried survival rate than that in a neutral environment (pH 6.5).

### 3.4. Mechanism of Influence of Surface Substances on Lyophilization Protectants of Stachyose

In order to determine the content of the stripped substances from the cell surface, the polysaccharide content of the supernatant was determined using phenol sulfuric acid, and the protein content was determined using the Kjeldahl method following the Chinese detection standard. The stripped surface substance mainly consisted of CPSs and SPs, which are (23.3 ± 2.4) ×10^−12^ and (13.0 ± 1.9) ×10^−12^ mg/CFU, respectively. CPSs accounts for the majority of surface substance. Studies have shown that the molecular weight of the protectant significantly affects the lyophilization protective effect of lactobacilli [26]. The molecular weight distribution of polysaccharides on the surface material of *L. casei* was determined by HPLC; the result showed that the molecular weight range of CPS was 10–300 kDa, which was significantly higher than that of trehalose and stachyose.

So as to determine the reason for the lyophilization survival rate differences between the *L. casei* with different amounts of surface substances, Fourier infrared spectrometry was applied to determine the bond energy to explain the differences between *L. casei* with different amounts of surface substance. The results are presented in Table 1 and Table 2. The lyophilized powder of *L. casei* FJSWX3-L3 fermented by arabinose contained a higher intensity hydrogen bond absorption peak (3200–3400 cm^−1^). In contrast, the intensity of the hydrogen bond absorption peak produced by *L. casei* FJSWX3-L3 bacterial powder in the MRS medium during the scanning process was weaker.

## 4. Discussion

The effect of surface substances on the freeze-dried survival rate of *L. casei* and its mechanism of action were investigated. After collecting the bacterial sludge, the stripping conditions of the surface substances were optimized to ensure that the bacteria were not damaged. The bacterial sludge was resuspended in 1 M NaCl solution. Due to the high osmotic pressure, the bacteria shrunk, and the gap between the surface substance and the cell wall increased. The surface substances were more likely to be broken and dissolved under ultrasonic cavitation, and the survival rate decreased with increasing ultrasonic time [23]. As high ultrasound frequency and excessive ultrasound time release a significant amount of heat, leading to a decrease in cell activity and even the rupture and death of cells [27], the maximum ultrasonic power and ultrasonic time (81 W and 18 min, respectively) occurred when cell activity was maintained without loss. The freeze-dried survival rate of *L. casei* before and after stripping the surface material was compared under optimal stripping conditions. The results showed that the freeze-dried survival rate of *L. casei* after stripping the surface material was lower without the protective agent. In contrast, the freeze-dried survival rate of *L. casei* with trehalose and fructose was higher (Figure 1), indicating that the production of surface substances could indeed improve the stress resistance of the strain without the addition of protective agents [12]. The survival rate of strains with surface substances was significantly higher than that of strains stripped of surface substances; however, when trehalose and stachyose were used as cryoprotectants, the existence of surface substances affected the protection of strains by cryoprotectants.

CPSs bind tightly to bacteria through divalent ions; therefore, divalent ions are essential in forming bacterial capsules [22]. As a cationic chelator, EDTA traps divalent ions between CPSs and bacterial cell walls during fermentation, allowing CPSs to dissociate. Appropriate use of EDTA could significantly reduce the production and yield of surface substances produced by the myxomycetes strain [23,28]. Based on these properties of EDTA, we investigated how the surface substances produced by the strain under the protection of trehalose and stachyose affected the survival rate during lyophilization. The results showed a negative correlation between the yield of surface substances and the survival rate during lyophilization (Figure 2). Therefore, on the premise that the growth efficiency of lactobacilli is not affected in lactobacilli with high surface substance content, the appropriate amount of EDTA could be added during the culture process to reduce surface substance production, to improve the lyophilization efficiency of fermentation and production efficiency.

Many researchers have attempted to optimize protective agents to improve the survival rate of freeze-dried bacteria, and more studies are focusing on improving bacterial resistance to improve the survival rate of freeze-dried bacteria, such as acid, low-temperature, and thermal stress. However, bacteria in the growth process inevitably produce surface materials, and the impact on freeze-dried bacteria survival has been neglected. It has been shown that in addition to strain differences and extraction methods, fermentation conditions, such as the carbon source [17], nitrogen source, carbon-to-nitrogen ratio, and pH, could also affect the production of surface substances [14]. In this study, the surface substances content and lyophilization survival rate of *L. casei* FJSWX3-L3 under different culture conditions were studied to analyze the specific relationship between them. The results showed that a low carbon-to-nitrogen ratio (4:3), low pH (5.0), and the use of pentose as the carbon source [19] could effectively reduce the production of surface substances by the bacterium, which was one of the methods to improve its survival rate and the number of live bacteria per unit mass of lyophilized powder when a polysaccharide was used as the lyophilized protectant. However, it remains to be studied and discussed whether these fermentation conditions cause other changes in the structure of lactobacilli and whether the survival rate of lyophilization could reach a peak level when lactobacilli does not produce surface substances at all.

An increasing number of experimental results [29,30] show that after being mixed with lactobacilli, the small-molecule carbohydrate protector forms hydrogen bonds with the cell membrane of lactobacilli after lyophilization and maintains its integrity and fluidity, which is the main reason for the high survival rate of lyophilized lactobacilli using small-molecule sugars as protective agents [31,32]. In addition, trehalose and stachyose can form a high-viscosity, low-fluidity glassy medium during the lyophilization process, which can combine with the cell membrane through the cell wall and replace the hydrated layer to protect the active cellular proteins when the protein hydration layer is disrupted [18,27]. The molecular weight, composition, and hydrogen bonding results of the surface substance with arabinose as the carbon source were analyzed to determine the mechanism by which surface substances affect the protective effect of stachyose. The results showed that most of the surface substances produced by *L. casei* FJSWX3-L3 during fermentation were CPSs as large molecules with significantly higher molecular weights than stachyose and trehalose may not protect bacteria as well as small-molecule carbohydrate protectants during freeze-drying [26]. This may occur because small-molecule sugars are less affected by steric hindrance and can form more hydrogen bonds [32]. The powder of *L. casei* FJSWX3-L3 obtained from fermentation when arabinose was used as the carbon source contained higher-intensity hydrogen bond absorption peaks, indicating that the excessive production of surface substances could affect the aggregation and action of trehalose and stachyose in the interstices of the membrane walls.

## 5. Conclusions

This study reports the effects of surface substances on the freeze-drying survival rate of *L. casei*. The results showed that excessive production of surface substances was not beneficial to the survival of *L. casei* during the lyophilization. The optimized cryoprotectant was much better than the surface substances of *L. casei* with respect to anti-freeze protection. In addition, this study showed that by changing the fermentation conditions, such as using arabinose as the carbon source or using EDTA as the chelating agent, the production of surface substances of *L. casei* could be effectively reduced, thereby improving its lyophilization survival rate. Excessive production of substances affects the strength of hydrogen bonds between polyhydroxyl protective agents, such as stachyose and trehalose, and the bacterial cell membrane. A small amount of surface substances could significantly affect the number and strength of hydrogen bonds formed between the bacteria and the protective agent and significantly affects the lyophilization survival rate of lactobacilli. Trace surface substances could significantly affect the number and strength of hydrogen bonds formed between the bacteria and the protective agent and significantly affects the lyophilization survival rate of lactobacilli.

## Figures and Tables

**Table 1:** The bond energy of freeze-dried *L. casei* cultured in MRS medium.

Absorption Peak (cm^−1^)	Intensity
598.15	0.865
861.16	3.205
1053.09	0.036
1412.48	0.709
1654.86	0.141
2931.11	0.487
3399.74	0.036

**Table 2:** The bond energy of freeze-dried *L. casei* cultured in MRS medium with arabinose as the carbon source.

Absorption Peak (cm^−1^)	Intensity
562.55	0.746
860.27	1.603
927.38	9.613
1052.95	0.083
1245.17	0.745
1413.11	0.707
1654.71	0.198
2931.39	0.542
3351.32	0.533

## Data Availability

The data used to support the findings of this study can be made available by the corresponding author upon request.
